# Involvement of *microRNA-34a* in Age-Related Susceptibility to Oxidative Stress in ARPE-19 Cells by Targeting the Silent Mating Type Information Regulation 2 Homolog 1/p66shc Pathway: Implications for Age-Related Macular Degeneration

**DOI:** 10.3389/fnagi.2019.00137

**Published:** 2019-06-13

**Authors:** Nianting Tong, Rong Jin, Zhanyu Zhou, Xingwei Wu

**Affiliations:** ^1^Department of Ophthalmology, Qingdao Municipal Hospital, Qingdao, China; ^2^Department of Pediatrics, Qingdao University Affiliated Hospital, Qingdao, China; ^3^Department of Ophthalmology, Shanghai Jiaotong University Affiliated Shanghai First People’s Hospital, Shanghai, China

**Keywords:** *microRNA-34a*, silent mating type information regulation 2 homolog 1, p66shc, age-related macular degeneration, oxidative stress, premature senescence

## Abstract

The aging retinal pigment epithelium and oxidative stress, mediated by reactive oxygen species (ROS) accumulation, have been implicated in the mechanisms of age-related macular degeneration (AMD). The expression level of the adapter protein p66shc, a key protein that regulates cellular oxidative stress, is relatively low under normal conditions because of the effects of silent mating type information regulation 2 homolog 1 (SIRT1) on the binding of fully deacetylated histone H3’ to the *p66shc* promoter region, thus inhibiting p66shc transcription and expression. The equilibrium between SIRT1 and p66shc is disrupted in the presence of various stresses, including AMD. As a major target gene, *SIRT1* is regulated by *microRNA-34a* (*miR-34a*), and overexpression of *miR-34a* results in significant inhibition of post-transcriptional expression of *SIRT1*. Furthermore, our recent studies demonstrated that *miR-34a* is significantly upregulated, accompanied by reduced tolerance to oxidative stress in hydrogen peroxide-induced prematurely senescent ARPE-19 cells. Moreover, the expression of SIRT1 is decreased, whereas that of p66shc is increased in these cells. Accordingly, *miR-34a* may play a key role in age-related susceptibility to oxidative stress in ARPE-19 cells by targeting the SIRT1/p66shc pathway, leading to AMD. In this review article, we discuss the functions of *miR-34a* in modulating the SIRT1/p66shc pathway in age-related conditions, including AMD.

## Introduction

Age-related macular degeneration (AMD), a leading cause of vision impairment in the elderly, is a multifactorial disease that involves age, gene variants of complement regulatory proteins, and smoking. With the increased aging of society, the number of patients with AMD is increasing, making this condition an important public health problem worldwide (Lim et al., 2012). AMD accounts for 54% of blindness in Caucasian Americana (Congdon et al., 2004), and a systematic review of data from an epidemiological survey of AMD prevalence found that the prevalence of any AMD ranged from 2.44% in people ages 45–49 years to 18.98% in people ages 85–89 years (Song et al., 2017). In a global meta-analysis, the overall prevalence of any AMD was 8.69% (Wong et al., 2014), and approximately 1.7 million people in the United States were affected by AMD, causing severe visual impairment in adults older than 65 years (Abbasi, 2018). Approximately 25.0% of eyes considered normal based on dilated eye examinations by primary eye care physicians (Neely et al., 2017); thus, the incidence of AMD is higher. The characteristic lesions of AMD are drusen, which are visible clinically in both the macula and retinal periphery (Mitchell et al., 2018). As AMD progresses, it can develop into two distinct forms of late AMD: “dry,” atrophic AMD, characterized by retinal pigment epithelium (RPE) senescence and geographic RPE loss, and “wet,” neovascular AMD, characterized by abnormal growth of choroidal new vessels (Zhu et al., 2009). Intraretinal or subretinal leakage, hemorrhage, and RPE detachments may occur with neovascular AMD, resulting in a rapid decline in vision. However, RPE atrophy or geographic atrophy may occur with late phase of dry AMD, resulting in a gradual vision decrease (Hallak et al., 2019).

Although the development of anti-vascular endothelial growth factor (VEGF) drugs has revolutionized the treatment of wet AMD (Martin et al., 2011), some patients show poor responses to these drugs. Additionally, there are few effective treatments for dry AMD. Therefore, current studies have focused on exploring the pathogenesis of AMD and identifying targets for early intervention and treatment of AMD.

Although the exact pathogenesis of AMD is not fully understood, a number of metabolic abnormalities have been shown to be associated with the development of this disease (Ding et al., 2009). For example, oxidative stress and oxidative stress-induced inflammation were found to initiate AMD in one study (Hollyfield et al., 2008).

In this review article, we provide a discussion of recent literature describing the mechanisms of AMD development, with a focus on RPE senescence, silent mating type information regulation 2 homolog 1 (SIRT1) signaling, and microRNA (miRNA) activity.

## Relationship Between RPE Senescence and AMD

Age is the most important risk factor for AMD (Curcio et al., 2009). More than 10% of older people over 80 suffer from advanced AMD (Smith et al., 2001). Indeed, with the increasing age, the organs and tissues of the whole body, including retinal tissue, showed decreased function. The RPE is a highly specialized epithelial cell layer with polarity that interacts with photoreceptors on its apical side and with Bruch’s membrane and the choriocapillaris on its basal side (Datta et al., 2017). The RPE functions in phagocytosis of the photoreceptor outer segment and thereby plays an important role in maintaining the normal physiological function of photoreceptors.

With aging and the gradual accumulation of environmental stresses, RPE cells gradually show dysfunction, including decreased phagocytosis, lipofuscin deposition, and drusen formation. These morphological and functional changes in the outer retina induced by RPE degeneration play an important role in the pathogenesis of AMD.

**Figure 1:**
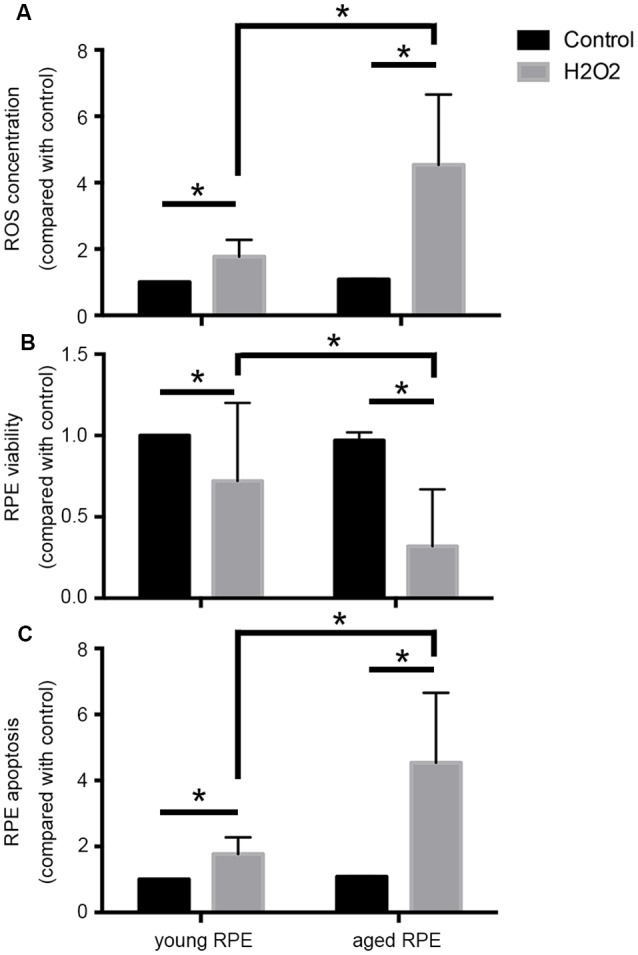
Aging induced susceptibility to oxidative stress in ARPE-19 cells. Aged ARPE-19 cells produced more reactive oxygen species (ROS; **A**), and cell viability decreased **(B)**, whereas apoptosis increased **(C)** significantly after hydrogen peroxide stimulation when compared with the effects in young ARPE-19 cells. *p < 0.05 significantly different when compared within two groups.

Notably, oxidative stress induces premature senescence in RPE cells, leading to an imbalance in VEGF and complement factor h, and may be a main player in the induction and progression of AMD (Marazita et al., 2016). As shown in Figure 1, in our preliminary experiments, we found that aged adult human retinal pigment epithelial (ARPE-19) cells produced more reactive oxygen species (ROS) and that cell viability decreased and apoptosis increased significantly after hydrogen peroxide stimulation when compared with the results in young cells, providing support for the age-related susceptibility of ARPE-19 cells to oxidative stress.

## Relationships Among *miR-34a*, RPE Senescence, and AMD Pathogenesis

miRNAs are small noncoding RNA molecules (~20–24 nucleotides in length) found in all cell types. These molecules lead to RNA silencing and post-transcriptional regulation of target gene expression, thus affecting cellular function and determining cell fate. Many miRNAs are differentially expressed in the circulation in patients with AMD (Ren et al., 2017). Additionally, significant changes in miNRA expression have been observed in retinal tissues from patients with AMD by bioinformatics and microarray technology. These miRNAs may be involved in the pathological processes of AMD by targeting downstream transcription factors. However, the specific regulatory mechanisms are still unclear (Berber et al., 2017).

As a p53-regulated miRNA, *miR-34a* was first discovered in studies of cancer. The activation of *miR-34a* promotes cell apoptosis and inhibits tumorigenesis. Recently, *miR-34a* has been shown to be closely related to cellular senescence in tissues. Notably, *miR-34a* is significantly upregulated in aged hearts and can promote the senescence of cardiac myocytes and decrease systolic function by inhibiting the expression of protein phosphatase 1 nuclear-targeting subunit and inducing telomere shortening (Boon et al., 2013). In contrast, *miR-34a* can also promote cellular senescence to maintain normal physiological function and avoid unlimited cell proliferation. Indeed, *miR-34a* regulates telomerase activity in hepatocellular carcinoma cells and promotes cellular senescence by targeting the FoxM1/c-Myc pathway (Xu et al., 2015). *miR-34a* is also a pivotal regulator of aging. For example, some drugs can regulate aging by affecting the expression of *miR-34a* and activating related signal pathways (Ye et al., 2016; Guo et al., 2017; He et al., 2017). Additionally, there was an age-dependent increase in *miR-34a* expression in the posterior pole of the mouse eye, and DNA damage in the mitochondria in retinal cells and RPE cells was found to be related to age and upregulation of *miR-34a* expression (Smit-McBride et al., 2014). Thus, *miR-34a* may play an important role in the pathophysiological mechanisms of cell senescence and apoptosis in the retina and in RPE cells in the aging eye.

**Figure 2:**
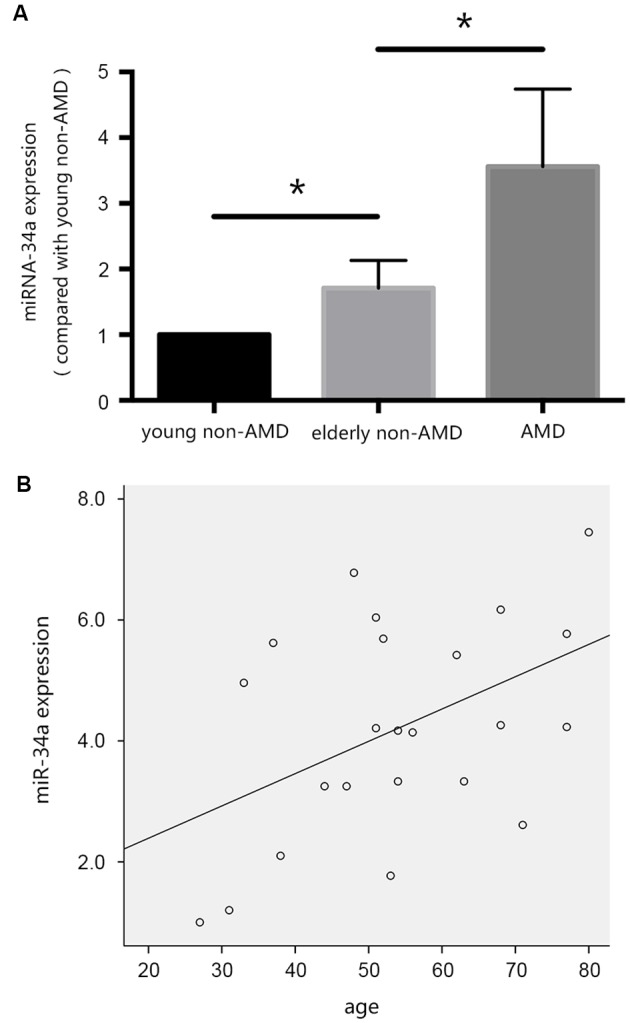
*miR-34a* may be involved in the pathogenesis of age-related macular degeneration (AMD). **(A)** The expression of *miR-34a* in the vitreous humor of the three groups of patients was increased. *p < 0.05 significantly different when compared within two groups. **(B)** Scatter plot showing the association between *miR-34a* expression and age. Note that there was a positive relationship between the expression of *miR-34a* and age (*r* = 0.457, *p* = 0.029).

In our previous studies, we evaluated the differential expression of *miR-34a* in the vitreous humor, collected during pars plana vitrectomy, from young patients without AMD, elderly patients without AMD, and patients with AMD. As shown in Figure 2A, the expression of *miR-34a* in the vitreous humor of the three groups of patients was increased, and there was a positive correlation between the expression of *miR-34a* and age (Figure 2B), as demonstrated by Pearson correlation analysis. These results indicated that *miR-34a* may be involved in RPE aging and the pathogenesis of AMD.

## The SIRT1/p66shc Pathway and Resistance to Oxidative Stress

SIRT1, as an NAD-dependent histone deacetylase in mammals (Vaziri et al., 2001), is a homolog of sir2 in lower organisms and plays important roles in various biological activities, such as the cell cycle, apoptosis, DNA repair, and gene silencing. The fundamental mechanisms through which SIRT1 regulates the expression of target genes involve histone H3 deacetylation of the promoter or enhancer (Oppenheimer et al., 2014; Hsu et al., 2016).

p66shc is widely expressed in vertebrates and is a key protein involved in intracellular regulation of oxidative stress and the life cycle. This protein induces cellular oxidative damage by producing a large number of ROS, mainly through the oxidation of cytochrome c in the mitochondria, and by inhibiting the elimination of ROS. Moreover, deletion of the *p66shc* gene can prolong the life span of mice by 30% *via* a mechanism involving enhancement of the resistance of mice to oxidative stress (Migliaccio et al., 1999).

Notably, SIRT1 can target the promoter region of *p66shc* (−508 to −250 bp), resulting in a decrease in the acetylation of histone H3 bound to this region. *p66shc* transcription is therefore inhibited, reducing tissue injury caused by oxidative stress. In acute ethanol-induced liver injury, the expression of p66shc was negatively correlated with changes in SIRT1 expression and decreased SIRT1 expression by RNA interference or nicotine significantly increased the expression of p66Shc (Tian et al., 2016). Therefore, the SIRT1/p66shc pathway may play important roles in blocking the effects of oxidative stress.

Many studies have shown that activation of SIRT1 may delay senescence in various tissues (Han et al., 2016; Guo et al., 2017; Hekmatimoghaddam et al., 2017), whereas inhibition of SIRT1 activity can cause premature senescence (Volonte et al., 2015), resulting in damage to cells caused by decreased tolerance of senescent cells to stress (Chuang et al., 2017). Additionally, the effects of SIRT1 on senescence are partly due to the regulation of *miR-34a* (Yang et al., 2013), although these effects have not been reported in retinal tissues and RPE cells. *miR-34a* was found to specifically bind with *SIRT1* mRNA in the 3′ untranslated region by bioinformatics prediction websites, such as pitcar, targetscan, Miranda, and microRNA.org.

**Figure 3:**
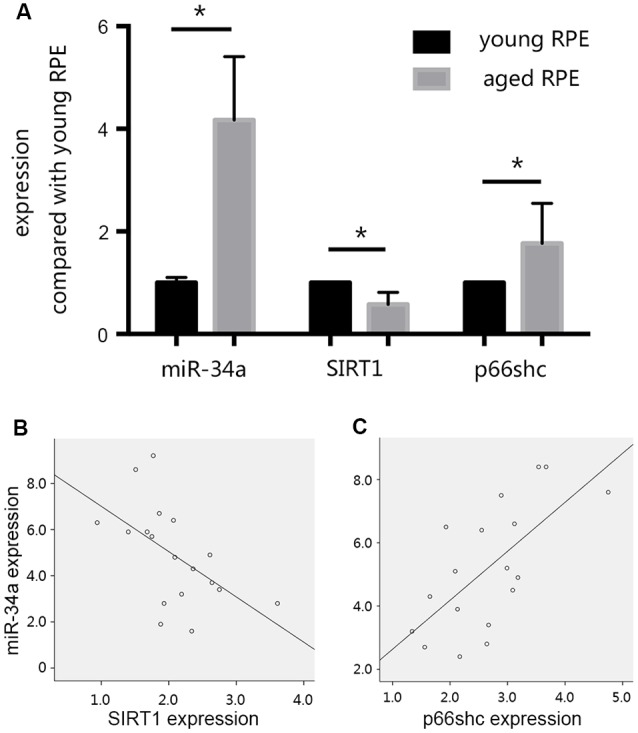
Changes in the expression levels of *miR-34a*, silent mating type information regulation 2 homolog 1 (*SIRT1)*, and *p66shc*. **(A)** The expression of *SIRT1* decreased, and the expression of *p66shc* increased in aged ARPE-19 cells, consistent with the increase in *miR-34a* expression. *p < 0.05 significantly different when compared within two groups. **(B)** Scatter plot showing the association between *miR-34a* expression and *SIRT1* expression in ARPE-19 cells. Note that there was an inverse relationship between these targets (*r* = −0.549, *p* = 0.018). **(C)** Scatter plot showing the association between *miR-34a* expression and *p66shc* expression in ARPE-19 cells. Note that there was a positive relationship between these targets (*r* = 0.666, *p* = 0.003).

In our preliminary experiments, we established an RPE premature senescence model by hydrogen peroxide stimulation as described previously (Aryan et al., 2016). ARPE19 cells were purchased from American Type Culture Collection (ATCC; Manassas, VA, USA). At passage 11, cells were seeded in 24-well plates at a density of 5 × 10^4^ cells/well, and were routinely cultured in 95% air and 5% CO_2_ at 37°C in DMEM/F12 medium containing 10% FBS, 50 units/mL penicillin, and 50 μg/mL streptomycin. After 24 h of incubation, the growth medium was removed and replaced with serum-free medium (SFM). The medium of selected wells was changed to SFM + 0.3 mmol/L hydrogen peroxide 24 h later. After 90 min of hydrogen peroxide treatment, the medium in selected wells was changed to 1 ml of DMEM + 10% FBS for the subsequent study on cell senescence. The effect of hydrogen peroxide on retinal pigment epithelial cell senescence was investigated by S-Beta Galactosidase staining 24 h after treatment with hydrogen peroxide. We found that when compared with young RPE cells, the expression of SIRT1 decreased, whereas the expression of p66shc increased in aged RPE cells, consistent with the increase in *miR-34a* expression (Figure 3A). A reverse correlation between the expression of *SIRT1* and *miR-34a* (Figure 3B) and a positive correlation between the expression of *p66shc* and *miR-34a* (Figure 3C) were found by Pearson correlation analysis, suggesting that *miR-34a* may directly inhibit the expression of SIRT1 in aged RPE cells, resulting in a decrease in the inhibitory effect on downstream p66shc and an increase in the expression of p66shc.

## A Hypothesis Regarding the Pivotal Role of *miR-34a* in Age-Related Susceptibility to Oxidative Stress by Targeting the SIRT1/p66shc Pathway

As described above, previous relevant studies and our preliminary experiments have led us to hypothesize that *miR-34a* may play important roles in age-related susceptibility to oxidative stress.

**Figure 4:**
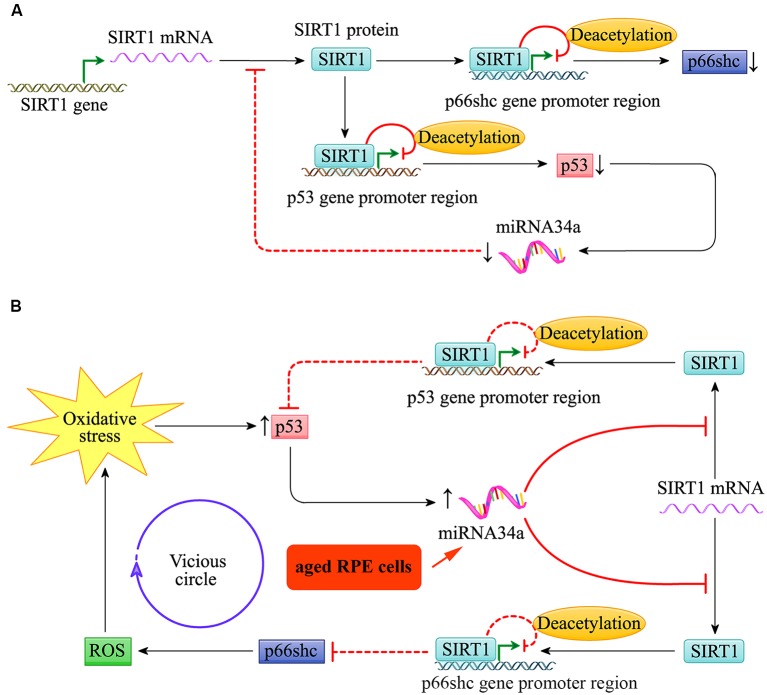
*miR-34a* regulated the SIRT1/p66shc pathway in young and aged retinal pigment epithelium (RPE) cells. **(A)** Schematic diagram for *miR-34a* and the SIRT1/p66shc pathway in young RPE cells. **(B)** Schematic diagram for *miR-34a* and the SIRT1/p66shc pathway in aged RPE cells.

Throughout life, the RPE cells are constantly challenged by high oxygen tension and exposure to photic stress, particularly in the macular region. As shown in Figure 4, the expression of the pro-apoptotic gene *p53* and *miR-34a* in young RPE cells was relatively low. The low expression of *miR-34a* reduces its inhibitory effect on the downstream target gene *SIRT1*, which can target the promoter regions of *p66shc* and *p53*, resulting in decreased acetylation of histone H3 bound to these regions. The transcription of *p66shc* and *p53* is therefore inhibited. Thus, the activities of p66shc and p53 in young RPE cells are relatively low, and the cells appear to be resilient to oxidative stress to a certain extent.

Additionally, as shown in Figure 4, the expression of *miR-34a* in the RPE increases with cell aging, and its effect on repression of *SIRT1* translation is enhanced. Then, the SIRT1/p66shc pathway is suppressed, and histone H3 deacetylation of p66Shc and p53 by SIRT1 is inhibited. The upregulation of p66shc induces oxidative stress by restoring excessive ROS and further induces the upregulation of *miR-34a* *via* the transcription factor p53. This results in a vicious circle of amplification for oxidative stress and leads to reduced resistance to oxidative stress in aged RPE cells.

## Conclusion

*miR-34a* may be involved in regulating susceptibility to oxidative stress by targeting the SIRT1/p66shc pathway during senescence in the RPE and the development of AMD. Targeting the inhibition of *miR-34a* may have implications in gene therapy for the early prevention and treatment of AMD. To test this hypothesis, further studies in mice with *miR-34a* knockout or overexpression are needed.

## Ethics Statement

The study was approved by Ethic Committee of Shanghai Jiaotong University Affiliated Shanghai First People’s Hospital, and patients provided written informed consent for participation in the study.

## Author Contributions

XW and NT conceived and designed the study. NT, RJ and ZZ performed the experiments and reviewed the literatures. NT and RJ wrote the article. XW, NT, RJ and ZZ reviewed and edited the manuscript. All authors read and approved the manuscript.

## Conflict of Interest Statement

The authors declare that the research was conducted in the absence of any commercial or financial relationships that could be construed as a potential conflict of interest.
